# Genome-Wide Identification, Phylogeny, and Abiotic Stress Response Analysis of *OSCA* Family Genes in the Alpine Medicinal Herb *Notopterygium franchetii*

**DOI:** 10.3390/ijms26115043

**Published:** 2025-05-23

**Authors:** Qi-Yue Zhang, Xiao-Jing He, Yan-Ze Xie, Li-Ping Zhou, Xin Meng, Jia Kang, Cai-Yun Luo, Yi-Nuo Wang, Zhong-Hu Li, Tian-Xia Guan

**Affiliations:** 1 Key Laboratory of Resource Biology and Biotechnology in Western China, Ministry of Education, College of Life Sciences, Northwest University, Xi’an 710069, China; 2022113027@stumail.nwu.edu.cn (Q.-Y.Z.); heexj13@163.com (X.-J.H.); 202310286@stumail.nwu.edu.cn (C.-Y.L.);; 2 Key Laboratory of Hexi Corridor Resources Utilization of Gansu, College of Life Sciences and Engineering, Hexi University, Zhangye 734000, China

**Keywords:** *Notopterygium franchetii*, *OSCA* gene family, genome-wide analysis, phylogenetic analysis, abiotic stress response

## Abstract

Hyperosmolality-gated calcium-permeable cation channel protein denoted as OSCA, which are mechanosensitive pore-forming ion channels, play a pivotal role in plants’ responses to abiotic stressors. *Notopterygium franchetii*, an endemic perennial plant species distributed in the Qinghai–Tibetan Plateau and its adjacent high-altitude regions, is likely to have undergone adaptive evolution in response to extreme abiotic stress conditions. The current study was conducted to characterize the genome-wide characteristics and phylogenetic evolution of the *OSCA* gene family in *N. franchetii* and identify its response patterns to drought and high-temperature stresses. We examined the gene family’s structural features, phylogenetic relationships, and response to abiotic stresses. The *N. franchetii* genome had 29 *OSCA* gene family members on 11 chromosomes. Subcellular localization showed they were mainly in the cell membrane, and a promoter cis-acting element study found that the *OSCA* gene family contained methyl jasmonate, abscisic acid, and various adversity and hormone response components. Under drought stress, most of the *NofOSCAs* genes showed a tendency to increase over time in the roots of *N. franchetii*, while in the aboveground parts, most of the *NofOSCAs* genes showed a tendency to increase and then decrease. The expression of different *NofOSCAs* genes in *N. franchetii* also showed alternating changes under high-temperature stress. Nine members of NofOSCAs were found to be linked to the PPI network, and these members were involved in membrane structure, transmembrane transport, and ion channel function. Our analysis of differential expression revealed that the expression of *OSCA* genes differed among the different *N. franchetii* tissues, with the roots exhibiting the highest average expression level, and many genes displayed tissue-specific high expression patterns. These results provided novel insights into the phylogenetic evolution and abiotic stress response mechanisms in the high-altitude medicinal herb *N. franchetii*.

## 1. Introduction

*N. franchetii* H. de Boissieu is a perennial medicinal plant native to China, mostly found in the Qinghai–Tibetan Plateau and the neighboring high-altitude regions. Rheumatoid arthritis, colds, fevers, joint and muscular aches, and other ailments can all be effectively treated with *N. franchetii*, the base plant of the *Notopterygium* medicinal herbs recognized in the Chinese Pharmacopoeia. In addition, it has antipyretic and analgesic properties, antibacterial and anti-inflammatory properties, antiarrhythmic properties, and myocardial ischemia protection [1,2]. According to recent pharmacological research, *N. franchetii* contains coumarin-like substances that can successfully lessen the inflammatory pain brought on by CFA [3]. *N. franchetii* and the same genus, *N. franchetii*, are closely related, according to phylogenetic analyses [4,5]. Significant population expansion events occurred during *N. Franchetii’s* evolutionary history, according to population genetics analyses [4,6], and research has indicated a significant correlation between the species’ adaptive evolution and species differentiation and the highly variable environmental and climatic oscillations at high altitudes [7] throughout the evolution of *Notopterygium*. The formation of distinctive medicinal components in this species may be largely due to the substantial enrichment of metabolic pathways like phenylpropanoid and flavonoid [8] biosynthesis in the extended gene family of *N. franchetii*. Furthermore, two genome-wide diploid duplication events unique to the Umbelliferae family occurred in *N. franchetii* after the genome triploid duplication event that real dicotyledonous plants share, according to evolutionary genomics investigations [9]. However, it is still unknown what molecular mechanisms underlie the growth, subfamily diversification, and functional redundancy or specialization of the gene families involved in resilience as a result of these replication events.

Generally, when environmental adaptations evolve throughout time, plants are subjected to a range of biotic and abiotic pressures [10]. Salt stress is one of the main stressors that cause the decline in growth and yield of nearly all terrestrial plants [11], while high osmotic stress brought on by drought and high temperatures can harm plants in a variety of ways, including water imbalance, growth slowdown, and leaves displaying varying degrees of wilting or even yellowing [12,13,14]. These effects can severely limit plant growth and reduce crop yield and quality [15]. High salt stress also lowers the rate of photosynthetic responses in leaves, impacts the synthesis of absorbed products, and alters the intensity of respiration [16,17]. It also impacts the fresh and dry weights of roots and stems, as well as the rate of plant leaf area expansion [18]. Because of the harsh temperature and climatic conditions in which it lives, *N. franchetii* may be strongly impacted by abiotic factors such as drought, low temperatures, and salt throughout its growth and development [19,20]. When calcium channels are activated, calcium ions, which are second messengers in the plant’s defense mechanism against unfavorable external environmental conditions, may rapidly increase in concentration. This creates a stressful calcium signal that further increases the plant’s resistance to deal with external abiotic stresses [21,22]. Calcium-permeable cation channel proteins (hyperosmolarity-gated calcium-permeable channels, OSCA) are hyperosmolar stress-sensing proteins (also called sensor proteins) that control Ca^2+^ concentrations to reduce the osmotic potential of plant cells, even though calcium signaling and calcium ion pathway proteins in plants are closely related.

Plant hyperosmotic stress is largely influenced by OSCA, the first calcium channel in plants that can detect external osmotic stress [12]. The osmoreceptor OSCA1 was discovered by the researchers using an objective forward-screening method based on plant in vivo calcium imaging after the calcium channel protein OSCA was discovered and reported in the model plant *Arabidopsis thaliana* in 2014 [23]. The study showed that OSCA1 proteins function as calcium channel proteins in response to abiotic stresses in plants [24], suggesting that calcium channels have an important role in plant responses to osmotic stresses. Three conserved functional structural domains have been identified in OSCA family proteins: RSN1_TM (pfam13967), PHM7_cyt (pfam14703, DUF4463), and RSN1_7TM (pfam02714, DUF221) [21]. RSN1_7TM was predicted to be the transmembrane region of the osmolarity-sensitive calcium-permeable cation channel, representing the seven transmembrane structural domain regions of calcium-dependent channels; RSN1_TM was predicted to be the first three transmembrane proteins; and PHM7_cyt was predicted to be the cytoplasmic domain of the integral membrane proteins [12]. The structural domain of RSN1_7TM was found to be able to mitigate the damage caused by osmotic stress to plants through osmoregulation [25] in the earlier systematic analysis and characterization of the *OSCA* gene family in reeling flower and land cotton (*Gossypium hirsutum*) [12,26], *A*. *thaliana*, tomato, and other model plants. Furthermore, the *OSCA* gene family’s promoter region plays a significant role in plants’ stress responses and has a large number of response components linked to stress response [12].

The majority of the 23 *OSCA* members found in tobacco (*Nicotiana tabacum*) exhibited upregulated expression under salt and drought stress, and the promoter region of this gene family contained the greatest number of drought-responsive elements [25]. These members were expressed in all tissues and organs and at all times. A strong response to salt stress was seen in 36 *OSCA* genes in ginger [12]. Additionally, it was shown that rice and soybeans are ten and thirteen members of the *OSCA* gene family, respectively, and are highly sensitive to salinity and drought stress [27]. Similar to rice, OsOSCA1.1 reacted selectively to salt stress (100 mM NaCl) and hypertonicity (250 mM sorbitol) in roots, causing stomatal closure by activating calcium channels to preserve leaf water balance. The overexpression lines were noticeably more drought-resistant than the mutants, which showed decreased survival and increased stomatal conductance. While OsOSCA2.1/2.2 can function as hypo-osmotic receptors and mediate extracellular water signals during pollen germination, which are then converted to calcium oscillations to promote pollen tube protrusion, OsOSCA1.1 improves osmoprotection by controlling downstream genes through the ABA signaling pathway (ABRE element) and calcium-dependent transcription factors [28]. The double mutant lacks hypotonic-induced calcium signaling and has a lower pollen germination rate [29].

By triggering calcium signaling and the antioxidant system at low temperatures (4 °C), GmOSCA1.1 can reduce oxidative damage in soybeans. GmOSCA1.1 formed a regulatory network with transcription factors, including CAMTA2 and WRKY33, and its expression was substantially greater in Huaxia 3 (cold-tolerant variety) than in Heihe 43 (sensitive variety). To encourage the accumulation of unsaturated fatty acids and preserve the fluidity of the cell membrane, the *GmOSCA* gene can be co-expressed with genes involved in fatty acid metabolism. While GmOSCA3.2 increased flooding resistance by controlling the ethylene signaling pathway and aeration tissue formation genes, it was upregulated in roots that had been waterlogged for 72 h [30]. The *OSCA* gene family’s precise number of members, chromosomal location, and sequence variation within the *N. franchetii* genome, however, remain unclear. Even though the OSCA family’s conserved structural domains (RSN1_TM, PHM7_cyt, and RSN1_7TM) are known, it is still unclear if the transmembrane region (RSN1_7TM) of the OSCA protein of the high-altitude medicinal plant *N. franchetii* contains any particular amino acid mutations that are suited to harsh conditions. Phylogenetic comparisons of closely related model plants and thorough investigation of the *cis*-acting elements in the promoter region of the *OSCA* gene of *N. franchetii* are lacking. Furthermore, whether the *OSCA* gene contributes to the distinct anti-stress strategy of the alpine plant *N. franchetii* in comparison to other plants has not been empirically confirmed.

Therefore, the *OSCA* gene family of the Chinese endemic alpine medicinal plant, *N. franchetii*, was the focus of the current study. The first goal was to clarify the structural characteristics of the gene family, such as member identification, conserved functional domains, and the distribution of stress-responsive cis-elements in the promoter region; the second was to reveal the gene family’s phylogenetic relationship, analyze evolutionary divergence from its relatives, classify its subfamilies by creating a phylogenetic tree, and investigate how some of the *OSCA* genes responded to drought and high temperatures. Additionally, by building a phylogenetic tree to examine evolutionary differentiation and subfamily classification, we were able to uncover the evolutionary relationship between OSCA and its relatives. We also identified the response patterns of certain *OSCA* genes to drought and high temperatures, as well as the changes in their expression levels over time. This study’s findings will offer crucial foundational information for a more thorough investigation of the mechanism of OSCA action, as well as crucial information for identifying the molecular underpinnings of stress tolerance in alpine medicinal plants. They will also offer potential target genes and a theoretical framework for the molecular breeding of disease resistance in medicinal plants.

## 2. Results

### 2.1. Identification of OSCA Gene Family and Physicochemical Property Analysis of N. franchetii

The *N. franchetii* genome contains 29 members of the *OSCA* gene family, which are dispersed over 11 chromosomes. These members are referred to as *NofOSCA1~NoOSCA29* according to their chromosomal positions (Table 1). The family proteins, according to physicochemical analyses, had a mean length of 732 aa and molecular weights that varied significantly between 15.9 and 140.3 kDa; 26 members (90%) were basic and had isoelectric points greater than 7, while 18 members (62%) were unstable proteins with transmembrane structural domains ranging from 0 to 12. There is clear potential for functional differentiation; 26 members (90%) are basic and have isoelectric points larger than 7, whereas 18 members (62%) are unstable proteins with instability coefficients greater than 40. The number of transmembrane structural domains varies from 0 to 12. NofOSCA1 and NofOSCA11 were specifically localized in chloroplasts, NofOSCA09 was localized in the cell wall, and NofOSCA05 and three other members were localized in the nucleus. The predicted subcellular localization revealed that the majority of the members were located in the plasma membrane. This suggested that the subcellular distribution of this family was diverse and that they may be involved in biological processes in various cellular compartments.

### 2.2. Secondary and Tertiary Structure Analysis of OSCA gGene Family Proteins in N. franchetii

The α-helix, elongated strand, β-turn, and irregular curl are the four components that make up the OSCA proteins of the *N. franchetii*, according to the projected secondary structures of 29 NofOSCAs proteins (Table 2). Of these, the percentages of the α-helix (25.64%~59.08%) and irregular curl (29.03%~50.88%) were higher than those of the extended chain (8.28%~18.81%) and β-turn (0.77%~4.66%). NofOSCA11 exhibited the lowest percentage of α-helix and the highest percentage of extended chain, β-turn, and irregular curl.

**Figure 1:**
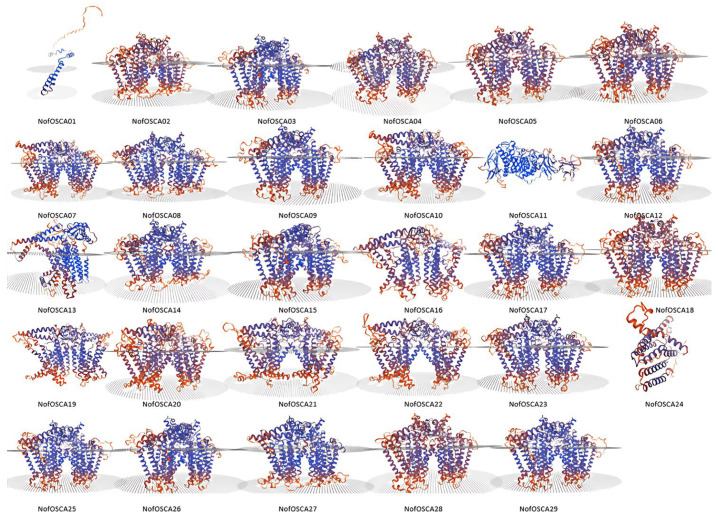
Results of three-dimensional structural analysis of *N. franchetii* OSCA proteins.

The majority of the *N. franchetii OSCA* gene family members have conserved tertiary structures that are comparable to those of OSCA proteins from other known species, which is consistent with the secondary structure predictions made by the SWISS-MODEL online tool for tertiary structure prediction of *N. franchetii* OSCA gene family proteins (Figure 1). The fact that NofOSCA1 and NofOSCA11 are mostly located in chloroplasts to carry out particular tasks may be the cause of their variances.

### 2.3. Phylogenetic Tree Analysis of N. franchetii OSCA Gene Family

**Figure 2:**
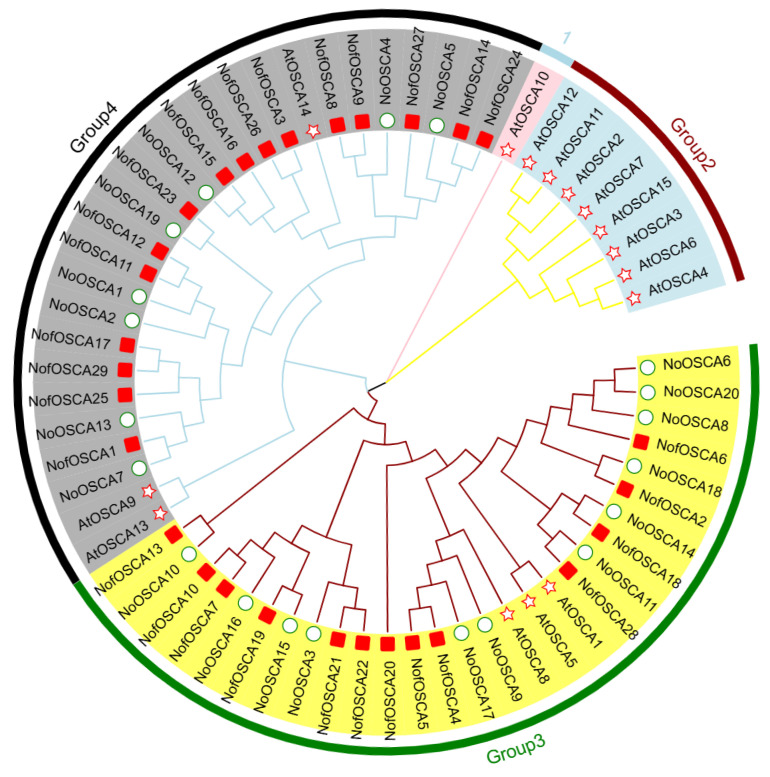
Phylogenetic tree of *OSCA* gene family in *N. incisum (circle)*, *N. franchetii (square),* and *A. thaliana (pentagram)*. The “pink lines” refer to Group 1, the “yellow lines” refer to Group 2, the “red lines” refer to Group 3, and the “blue lines” refer to Group 4.

This study downloaded the protein sequences of *A. thaliana* to uncover the evolutionary relationship of the *N. franchetii OSCA* gene family. After obtaining and identifying the *N. franchetii* and *N. incisum* protein sequences from the laboratory, the evolutionary tree was built using MEGA 11 (Figure 2). The results showed that the OSCA genes of the three species were separated into four subfamilies, with the *A*. *thaliana* OSCA family members concentrated in two subfamilies, Groups I and II, and only three members in Groups III and IV, respectively. This was consistent with the findings of previous evolutionary analyses of OSCA family members in *A*. *thaliana*. AtOSCA10, which had little in common with other OSCA members, inhabited Group I on its own. This is consistent with the results of previous evolutionary investigations of members of the OSCA family of Arabidopsis. The members of the *N. incisum* OSCA and *N. franchetii* families were primarily divided into two subfamilies, Groups III and IV. However, the distribution of members within these two subfamilies was not uniform; the *N. franchetii* OSCA family members were more widely distributed in Group IV, which had 16 members, and less widely distributed in Group III, which had 13. In Group III, the members of the *N. franchetii* OSCA family were more widely spread. Members of the *A*. *thaliana* OSCA family were more distantly connected to the *N. franchetii* OSCA family, although they were more homologous with certain other members of the *N. incisum* OSCA family.

### 2.4. Conserved Motifs, Conserved Structural Domains, and Gene Structure Analysis of N. franchetii OSCA Gene Family

**Figure 3:**
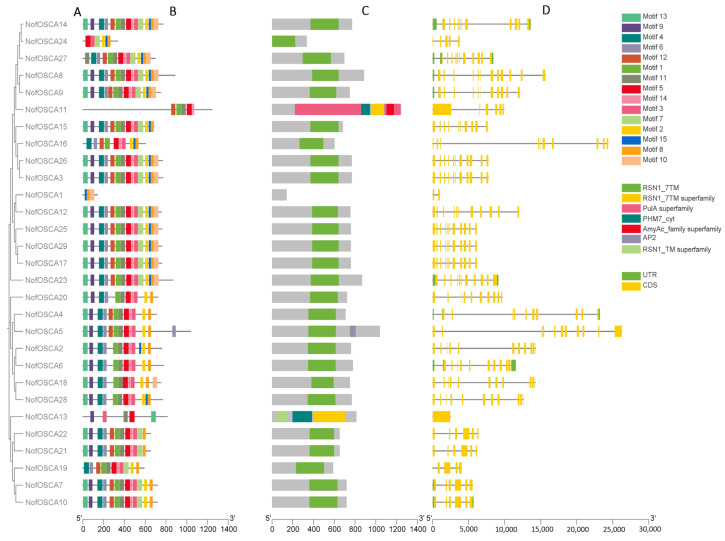
The phylogenetic relationships, distribution of conserved motifs, domain prediction, and gene structure analysis of *N. franchetii OSCA* genes. (**A**) A phylogenetic tree of 29 *NofOSCA* genes. (**B**) The distribution of conserved motifs in OSCA proteins. Fifteen conservative motifs in OSCA are displayed in different color boxes. (**C**) The domains of OSCA proteins. These denote the matching types that represent various confidence levels (specific matching and non-specific matching) and domain model ranges (superfamily and multi-domain). (**D**) *OSCA* gene structures. The description of exons and introns is obtained by adopting image tools. The yellow and green boxes and the green lines represent the non-coding region (UTR) and exons and introns, respectively.

Figure 3 displays the conserved motifs, conserved structural domains, and gene structure of NofOSCAs. First, the sequences of 29 protein conserved domains of NofOSCAs were subjected to phylogenetic analysis. RSN1_7TM, the PFK superfamily, and the PKc-like superfamily were the three structural domains that were screened for NofOSCAs. All members of the *N. franchetii* OSCA family, except for NofOSCA1, contained RSN1_7TM; that is, all other members had OSCA conserved structural domains, and RSN1_7TM was predicted to contain OSCA conserved structural domains. The transmembrane region of osmotic pressure-sensitive calcium-permeable cation channels is predicted to be RSN1_7TM, which represents the seven transmembrane domains of calcium-dependent channels. This suggests that the 28 *N. franchetii* OSCA family member proteins should continue to function as osmotic pressure-sensitive calcium-permeable cation channels, as all other members contain OSCA conserved domains. Members have structural domains that are conserved by OSCA. Using MEME online software (suite 5.5.8), up to 15 conserved motifs were found in the OSCA proteins of *N. franchetii* species (Table S1). The results were verified by comparing them with the conserved structural domains. Except for NofOSCA1, the number of exons in the NofOSCA family members ranged from 2 to 19, according to the findings of the gene structure study of the OSCA family members. The remaining members of the subfamily essentially have all of the conserved motifs, and they are organized in a similar order, except NofOSCA1, NofOSCA11, NofOSCA13, and NofOSCA24, which have short, conserved motifs. Based on the motif composition analysis, it is hypothesized that Motifs 5 and 14 are closely related to the RSN1_7TM structural domain because they are present in all members except NofOSCA1, and their positions in the sequence are similar to that of RSN1_7TM. Additionally, Motifs 1 and 11 are present in the majority of OSCA family members, suggesting that Motifs 15 and 10 are primarily found in one subfamily and are thought to be related to the specific functions of members of this subfamily. Furthermore, it is assumed that NofOSCA2, NofOSCA6, NofOSCA18, and NofOSCA28 may have been specialized and may have rejected the sequence corresponding to Motif 12 during the evolutionary process because they all lack Motif 12 and have a high degree of homology. It was assumed that the protein structure of *N. franchetii* OSCA was highly conserved because the types, locations, and sequences of the conserved motifs of the members of the *OSCA* gene family were quite consistent. Functions may be similar, but not identical, between subfamilies.

### 2.5. Analysis of Cis-Acting Elements of the OSCA Family of N. franchetii

**Figure 4:**
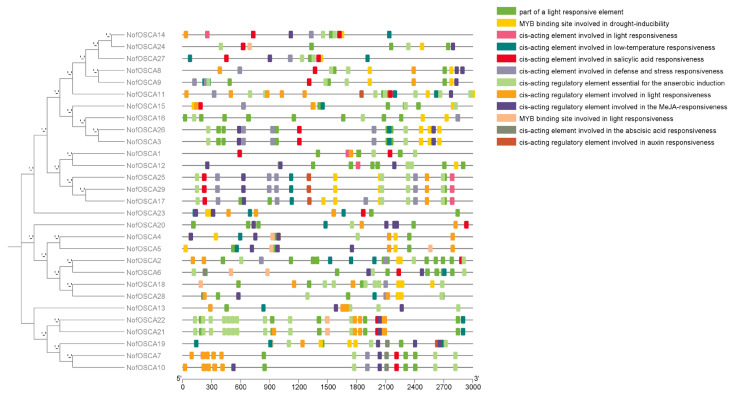
Analysis of *cis*-acting elements of the *OSCA* gene family of *N. franchetii*.

A variety of *cis*-acting elements, including methyl jasmonate-responsive, abscisic acid-responsive, drought-induced, low-temperature-responsive, resistance-responsive, and growth factor-responsive elements, were found in the promoters of the OSCA gene family members, according to the cis-acting element prediction of the 3000 bp base sequence upstream of the start codon of the *OSCA* gene (Figure 4). Each of these members had at least nine *cis*-acting elements. Six of the twenty-one members have three of the MYB binding sites implicated in drought inducibility. The majority of the *N. franchetii* OSCA family members are thought to be capable of responding to drought stress in different ways. It is hypothesized that OSCA family proteins are crucial for *N. franchetii’s* ability to adapt to low temperatures in highland and high-altitude mountainous regions because 22 members have *cis*-acting regulatory elements involved in low-temperature responses, and three of the members have three of these elements. MeJA responsiveness is mediated by *cis*-acting regulatory elements found in 25 members of the OSCA family. It is thought that these members are able to detect changes in the concentration of MeJA and bind to particular transcription factors, which in turn activate the expression of a number of defense-related genes and increase the resistance of plants to stressors. This is because MeJA levels in plants rise quickly when they are exposed to biotic or abiotic stresses. The *N. franchetii* OSCA family genes are thought to be responsive to variations in light intensity and time since 22 members contain light-responsive *cis*-acting regulatory elements, and there are eight of these upstream of *NofOSCA10*. The study of *NofOSCAs’* promoter *cis*-acting elements showed that, even within the same subfamily, the genes of various *NofOSCAs* members had considerably diverse promoter *cis*-acting elements. Adversity response elements and resistance were present in the great majority of *N. franchetii OSCA* gene family members. It is hypothesized that its *OSCA* family may be closely related to various types of stresses.

### 2.6. Chromosomal Localization and Covariance Analysis of the OSCA Gene of N. franche-tii

**Figure 5:**
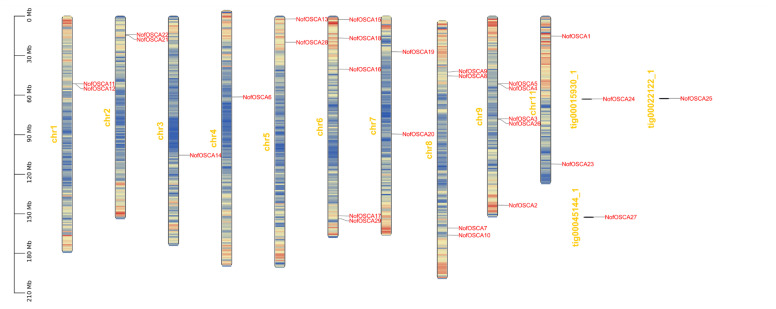
Chromosomal localization of *N. franchetii OSCA* genes. the “red words” refer to gene names, while the “yellow words” denote chromosome names and Contig numbers.

**Figure 6:**
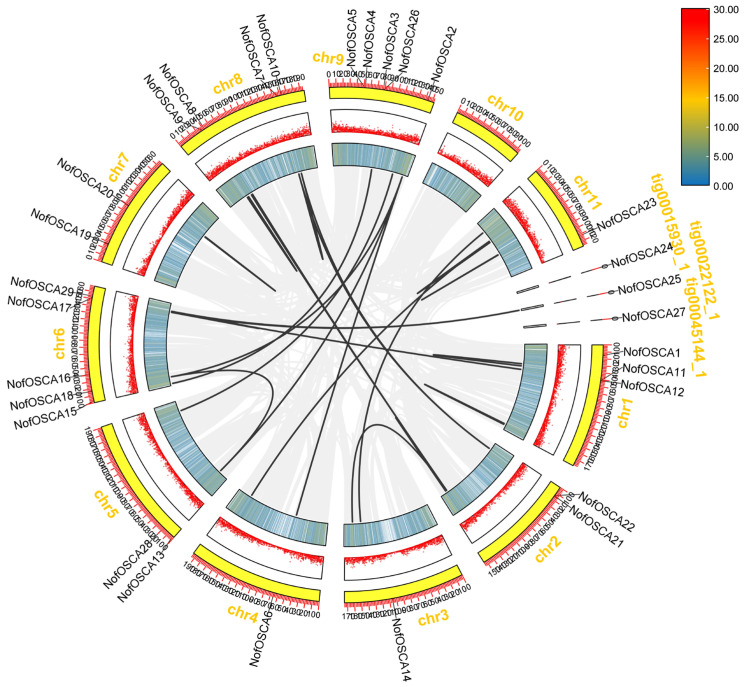
Results of covariance analysis of *N. franchetii OSCA* genes.

The chromosomes Chr1, Chr2, Chr3, Chr4, Chr5, Chr6, Chr7, Chr8, Chr9, Chr10, tig0022122-1, tig00015930-1, and tig00045144-1 contain the 29 *OSCA* genes of *N. franchetii* (Figure 5). The greatest number of genes was found on chromosomes Chr6 and Chr9, with five genes, followed by four on Chr8, and two on Chr1, Chr2, Chr5, Chr7, and Chr11. Each of the other chromosomes carries a single gene. Fifteen OSCA family members were involved in fragment duplication events between *N. franchetii OSCA* paralogous homologous genes, according to an intraspecific covariance analysis of *N. franchetii* (Figure 6). This indicates that the *N. franchetii OSCA* gene family contains a greater number of intraspecific covariance genes.

### 2.7. Ka/Ks Calculation

We calculated Ka, Ks, and Ka/Ks for 12 homologous gene pairs among *NofOSCAs* in order to ascertain the type and degree of selection pressure duplicate gene pairs face. Neutral selection is generally defined as Ka/Ks = 1, positive selection as Ka/Ks > 1, and purifying selection as Ka/Ks < 1. All homologous gene pairs had Ka/Ks values smaller than 1, according to the results (Table S2). Eleven of them had a Ka/Ks value below 0.50, while only NofSOSCA8/NofOSCA9 had a value above 0.5, suggesting that they may be undergoing a transition to selective pressure or may be the target of positive selection in certain unique situations. As a result, during the evolutionary process of selection, they were all subject to purifying selection, and even the great majority of them were subject to strong purifying selection.

### 2.8. GO, KEGG Enrichment, and PPI Networks Analysis of N. franchetii

**Figure 7:**
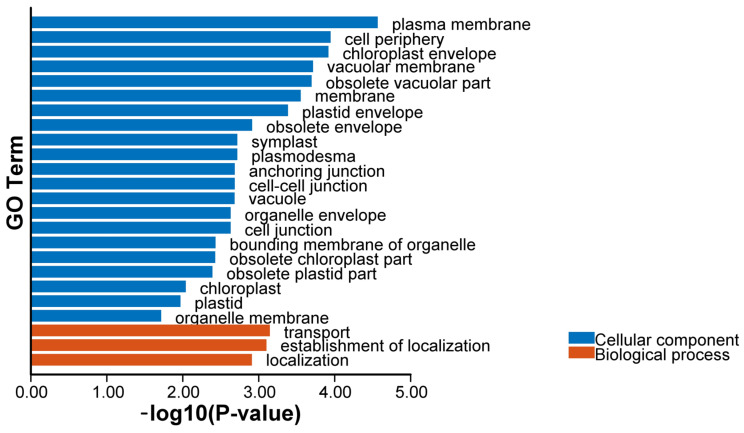
Results of GO enrichment of the *OSCA* gene family in *N. franchetii*.

The results of GO enrichment analysis (Figure 7, Table S3) showed that 29 genes were widely involved in the functional annotation related to cellular components and biological processes. The genes were substantially more abundant in membrane structures, including the organelle envelope, cell membrane, chloroplast periplasm, plasma membrane, and cell periphery, among other cellular components. With a corrected *p*-value of less than 0.002, the plasma membrane and membrane contained 10 and 11 hits, respectively, indicating that these genes were important for the makeup of cellular structure. The genes were mostly involved in transport, localization, and other biological activities. With a corrected *p*-value of 0.0039, five genes were found to be significantly enriched in transport. This suggests that these genes are involved in basic living functions like material transport and cellular localization, which are thought to be connected to the function of ion channels in the *OSCA* family. Several cell component-related GO terms shared genes such as *NofOSCA19*, indicating that these genes are somewhat conserved in cell structure-related functions.

**Figure 8:**
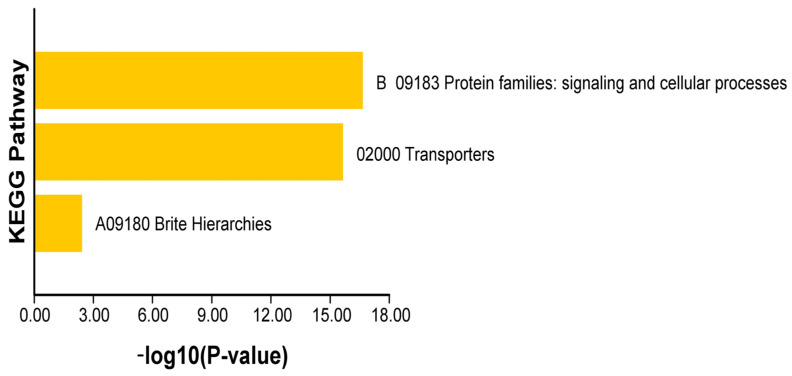
Results of KEGG enrichment of the *OSCA* gene family in *N. franchetii*.

With a *p*-value of 2.22 × 10^−16^ and an enrichment factor of 14.95 for the pathway “02000 Transporters”, the results of the KEGG enrichment analysis (Figure 8, Table S4) revealed that 29 genes (28 genes in the selected set, 27 hits) were significantly enriched in multiple pathways under the major category of “A09180 Brite Hierarchies”. This suggests that these genes are highly enriched in transporter-related functions and may be important for the transmembrane transport of ions, metabolites, etc. Together with the high hit ratios of the genes in protein families related to signaling and cellular processes, the “B 09183: signaling and cellular processes” pathway was also markedly enriched, indicating that these genes are also involved in the regulation of growth and development, cell signaling, and other critical biological processes. Due to their combined involvement in transporter function and signaling cell process-related pathways, the 27 genes may be essential in preserving the equilibrium of cellular material transport, controlling cell signaling, and governing fundamental life activities.

**Figure 9:**
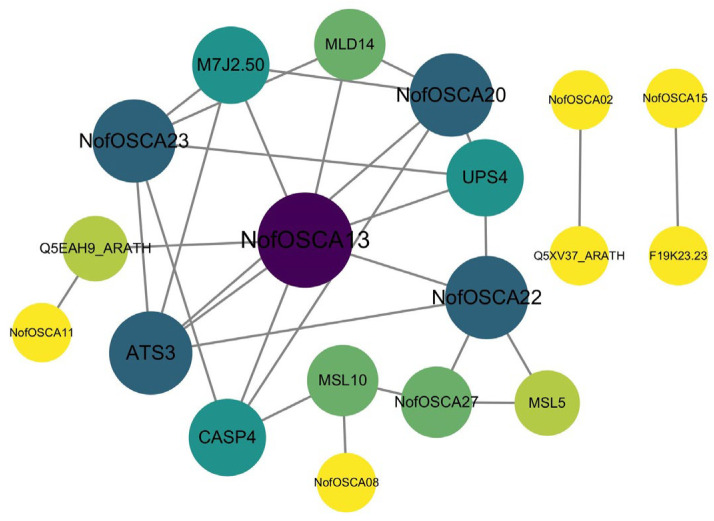
Protein–protein interaction analysis of OSCA proteins in *N. franchetii*. The color of the circle is darkened with the number of proteins with which it interacts.

Twenty-nine protein sequences of NofOSCAs were uploaded to the STRING database, and a protein interactions network relationship diagram of NofOSCAs was created in order to investigate the plant regulatory networks in which the *N. franchetii* OSCA gene family proteins may be engaged (Figure 9).

As shown in the figure, nine members, i.e., NofOSCA23, NofOSCA20, NofOSCA22, NofOSCA27, NofOSCA08, NofOSCA11, NofOSCA13, NofOSCA02, and NofOSCA15, were mapped into the PPI network. Some protein pairs had high-confidence interactions, like F19K23.23 with NofOSCA15 (0.926) and Q5XV37_ARATH with NofOSCA02 (0.899). The former involved unidentified proteins and CSC1-like calcium channel proteins, while the latter linked transmembrane proteins to members of the OSCA family, likely related to ion channel function or membrane structure. The interaction score of UPS4 (urotensin permease) with NofOSCA23 was 0.893, indicating a synergy between transmembrane transport and ion channels; CASP4 (involved in the formation of Casparian bands in the cell wall), NofOSCA08, and NofOSCA27 had a high interaction score with MSL10 (a mechanosensitive ion channel) that may be synergistic in root barrier function; and NofOSCA22 (a hyperosmotic gated cation channel) with NofOSCA13 and UPS4 indicate functional associations in osmotic stress response.

Experimental validation is necessary for lower confidence interactions like CASP4 with NofOSCA13. The high frequency of NofOSCA22 and NofOSCA23 indicates that they may form functional modules involved in mechanical stress response or calcium signaling; the interactions of transmembrane transport-related proteins with NofOSCAs, including UPS4 and MSL5/MSL10, suggest that cellular transmembrane substance transport is regulated synergistically; and the embryo-specific protein ATS3 interacts with multiple NofOSCA proteins, which may be involved in the regulation of ionic homeostasis during embryonic development.

### 2.9. Expression Analysis of N. franchetii OSCA Gene Under Drought and High-Temperature Stresses

**Figure 10:**
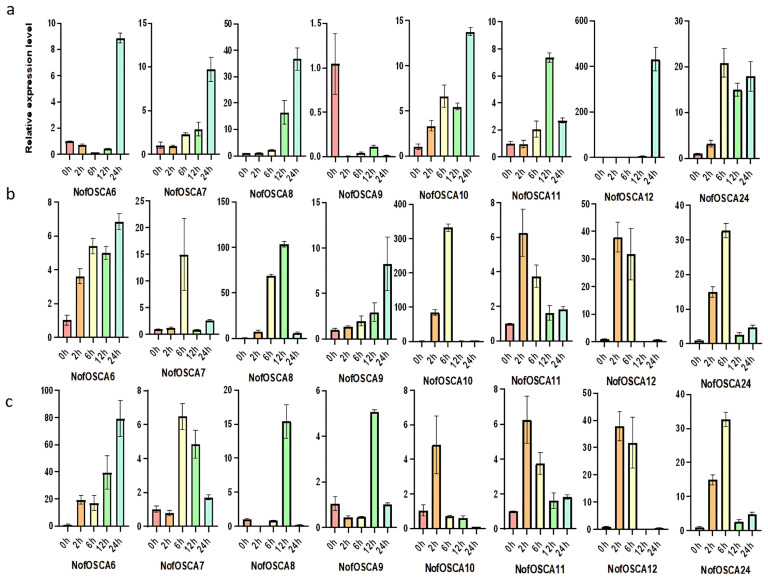
Expression analysis of *N. franchetii OSCA* genes under drought stress. (**a**) leaf; (**b**) stem; (**c**) root; internal reference gene: *NofActin8*; *n* = 3.

**Figure 11:**
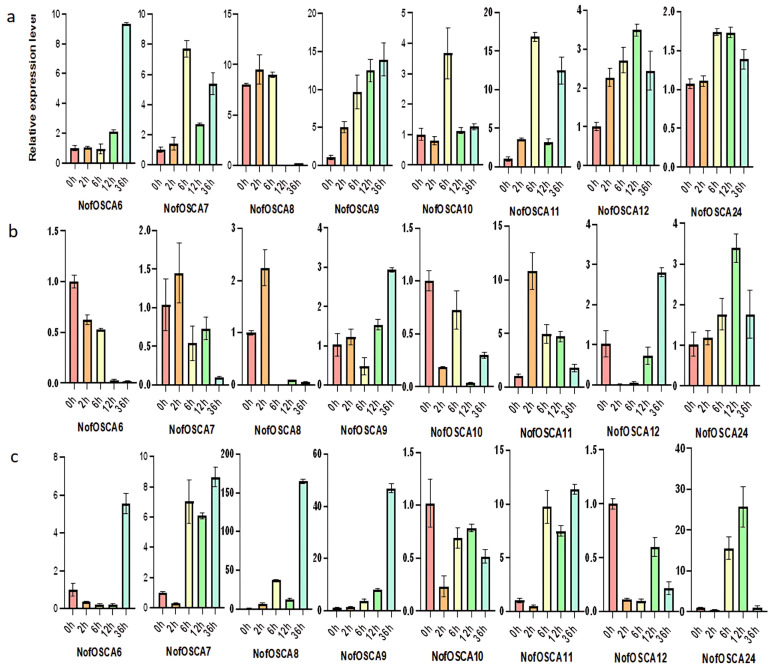
Expression analysis of *N. franchetii OSCA* genes under high temperature. (**a**) leaf; (**b**) stem; (**c**) root; internal reference gene: *NofActin8*; *n* = 3.

Three-year-old farmed *N. franchetii* plants with medicinal value were exposed to stress treatments in order to examine the reaction of the *N. franchetii OSCA* gene under environmental stressors such as drought and high temperatures. To replicate dryness and high temperatures, respectively, seedlings that had been re-sprouted for six weeks were put in an incubator set at 40 °C and 40% PEG 6000 solution. Based on the transcriptome sequencing data, the 29 *OSCA* genes in the pre-experimental samples were analyzed for their expression relative to the internal reference gene *NofActin8* and combined with the qRT-PCR pre-experiment results. After excluding genes with expression fluctuations of less than 1.5-fold, the eight genes with the highest average expression throughout the drought and high-temperature stresses were selected as the subjects of the experiment (Table S5). The degree of fluctuation in the relative expression of *NofOSCA* genes under various stress treatments was measured using error bars in the qRT-PCR findings shown in Figure 10 and Figure 11. For every time point in the experiment, three biological replicates were established. The error bars’ widths indicate the degree of dispersion among the replicate samples; a narrow error bar indicates high data reproducibility, while a wide error bar suggests some variation among the samples. The experiment’s *p*-values were tested to be less than 0.05, and the error bars between adjacent time points did not overlap.

The expression patterns of various *NofOSCAs* in roots, stems, and leaves under drought stress varied significantly; aboveground parts (stems and leaves) displayed a predominantly increasing trend followed by a decreasing trend, with *NofOSCA6/9* showing a decreasing trend overall, while *NofOSCAs* in roots showed a predominantly increasing trend and only *NofOSCA9* showed a decreasing trend, with the highest expression at 0 h. The most noticeable increase in expression over time was seen in *NofOSCA12; NofOSCA6* displayed a trend of a slight decline followed by a rapid rise; and *NofOSCA11/24* both had a tendency of an increase followed by a decline (Figure 10a). The most noticeable shift in expression in stems was displayed by *NofOSCA10*, which peaked at 6 h after trending upward and then downward. The remaining genes displayed an increasing and then declining pattern, peaking at different times, while NofOSCA6/9 displayed a steady increasing trend (Figure 10b). After 12 h, *NofOSCA8/9* in leaves grew rapidly, but *NofOSCA6* continued to increase, with the biggest change (Figure 10c).

Under high-temperature stress, the expression patterns of various *NofOSCAs* genes in roots, stems, and leaves varied by site; most genes in roots and stems displayed an increasing and then decreasing trend coupled with an alternating pattern of change, while more genes in leaves displayed an overall increasing trend and a small number of genes displayed an overall decreasing trend with a complex pattern of alternating change. In roots, *NofOSCA7/11* had a steady pattern of expression, with both alternating between rising and falling. *NofOSCA6* displayed the most pronounced continuous rise, while *NofOSCA8* showed a minor rise followed by a steep decline (Figure 11a). Alternating changes in *NofOSCA7/10* were observed in stems, where *NofOSCA11* exhibited the most notable increase, followed by a decrease, while *NofOSCA6* continued to decrease (Figure 11b). Alternating changes were observed in leaves in *NofOSCA8/10/12*, where *NofOSCA9* continued to rise and *NofOSCA7/11* showed a slight decline followed by a sharp rise (Figure 11c).

### 2.10. Expression Analysis of OSCA Gene Expression Among Different Tissues in N. franchetii

**Figure 12:**
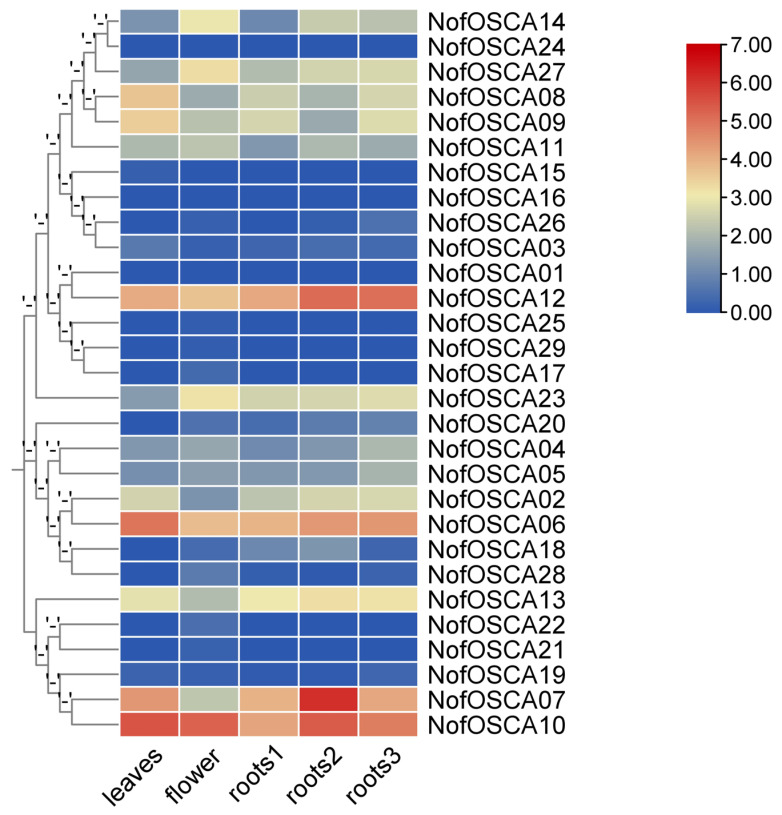
Gene expression heat map of *NofOSCAs* in different tissues of *N. franchetii*.

When transcriptome data from various *N. franchetii* tissues were analyzed, it was found that there were differences in gene expression between the tissues (Figure 12). Of these, root 2 had the highest average expression level and the highest degree of dispersion, suggesting that it may be involved in more active life processes and regulated by complex factors. This suggests that the physiological processes or functional associations between leaves and flowers may be similar, while the correlation between roots and other tissues was low, suggesting that the functions or regulatory mechanisms may be more distinct. Gene expression in leaves and flowers showed a strong link, indicating that these tissues might share physiological functions or associations. The number of highly expressed genes in all the tissues was 7, indicating that the various tissues may balance the number of highly expressed genes to ensure the basic activities of life. This was discovered when the 75% quartile of gene expression was used as the threshold for screening the highly expressed genes in each tissue.

In terms of transcriptome values, there were significant differences among the genes. *NofOSCA01* and *NofOSCA16* had values of 0 under multiple transcriptome types, which may indicate that the transcriptional activities of these genes were at a very low level in the corresponding tissues or were not detected. In contrast, *NofOSCA06* had the highest expression in leaves, *NofOSCA12* had the highest expression in roots, *NofOSCA18* and *NofOSCA20* displayed a trend of differential expression in various sub-tissues of the root system, and *NofOSCA07* was expressed significantly higher in roots than in other tissues, exhibiting an extremely high expression. *NofOSCA10*, on the other hand, was highly expressed in leaves and flowers, with relatively low expression in roots. Important candidate genes for the study of *N. franchetii’s* growth, development, and metabolic mechanism may be provided by these genes, which may also be regulated by tissue-specific regulation or play crucial roles in the functional differentiation of various tissues.

## 3. Discussion

Plants are exposed to a variety of osmotic challenges throughout growth and development, including cold, salinity, drought, and other abiotic stresses that happen at every stage of plant growth and development [31]. Plants may find it challenging to maintain proper cellular structure and ionic equilibrium as a result of these abiotic stressors. As a second messenger, calcium ions are crucial for signal transduction and the plant’s reaction to environmental stress. Plants can respond to adverse stress by controlling intracellular Ca^2+^ content through Ca^2+^ channels in response to external stimuli [32]. The highly conserved structural domains of the OSCA proteins of ion channels are crucial for controlling plant signal transduction. It has been demonstrated that members of the *OSCA* gene family are found in the plasma membrane and function as osmotic pressure sensors mediating intracellular osmotic pressure changes [33,34]. These members can respond significantly to the induction of adversity stress, and the expression of this gene can significantly increase the plant’s resistance to adversity [35,36]. The majority of research on the *OSCA* gene family in plants has concentrated on model plants and rice [37]. The three proteins of the OSCA family and DUF221, a cytoplasmic region of integral membrane proteins, are found in a wide range of plant OSCA family members. The three conserved structural domains of OSCA family proteins are RSN1_7TM (DUF22), which acts as a calcium channel in osmotic sensing; RSN1_TM, which is linked to vesicle transport (cytotoxicity); and PHM7_cyt (DUF4463), which typically occurs before and after RSN1_7TM. A variety of aspects of plant growth, development, response to abiotic stressors, and hormone transduction pathways are regulated by proteins containing the DUF221 domain [38], which is present in members of the *OSCA* gene family and contains six to seven transmembrane structural domains, such as those found in Arabidopsis, soybean, and rice [21,39].

Several crops have been found to have the *OSCA* gene family, with 15 classified into four subfamilies in Arabidopsis [23], 11 in rice [27], 12 in maize [40], 16 in pears [37], 13 in mung bean (*Vigna radiata*) [41], 16 in kiwi [42], 12 in tea [43], and 13 in thistles and clover [44]. The number of *OSCA* genes varies among species. Thirteen in tribulus clover [44], twelve in tea tree [43], and sixteen in kiwifruit [42] were categorized into four subfamilies. Similar to those found in wheat and ginger, a total of 29 members of *NofOSCA* were found in *N. franchetii*, which were separated into 3 subfamilies [12,45]. This may be due to the size of the plant’s genome and the quantity of tandemly duplicated genes, which may indicate that gene deletion and retention may be linked to specific functions during plant evolution [46].

Consistent with the findings of earlier research, every member of the *N. franchetii* OSCA proteins in this investigation possesses the SRN1_7TM structural domain. The proteins of the majority of the *N. franchetii* OSCA family were primarily found in the plasma membrane, according to the predicted subcellular localization of the family’s proteins. This outcome is in line with localization findings in plants such as hairy poplar (*Populus tomentosa*), soybeans, and pepper (*Capsicum annuum*) [21,47,48]. The plasma membrane contains OSCA proteins, which are essential for the early stages of the hyperosmotic stress response [49]. The promoter region of *NofOSCA*s was shown to contain *cis*-elements for several stress and hormone responses, including light [50], drought [51], abscisic acid [52,53], and methyl jasmonate [54]. It implies that *OSCA* genes might play a role in a variety of stress and phytohormone response mechanisms that are linked to plant growth and development as well as stress tolerance [45]. According to the phylogenetic tree, the NofOSCA family could be categorized into three groups. This finding is in line with the classification of *OSCA* gene families in soybean, wheat, and sunflower (*Helianthus annuus*) [21,55,56]. The majority of the *N. franchetii* OSCA members have conserved motifs arranged in a similar manner and order, and their proteins are highly conserved. However, the number of exons and conserved motifs in various subfamilies varies greatly. The exon and intron dynamics of a gene can reflect the evolutionary process of the gene family [57]. The *N. franchetii* OSCA family members were more distantly connected to the OSCA family members of *A. thaliana* and more homologous to some other members of the *N. incisum* OSCA family, according to the results of gene family phylogenetic connections. Ginger OSCA exhibited more distant connections with soybean and Arabidopsis and greater similarities with certain members of the rice family in the evolutionary study of its OSCA gene family [12]. According to the findings of earlier research, OSCA gene families, including those found in rice and Arabidopsis, are linked to the control of adversity [27,35]. Although no *cis*-acting element for drought stress response was revealed in the promoter region of the *CaOSCA11* and *CaOSCA14* genes, a study on the *CaOSCAs* gene family of pepper indicated that these genes could still induce a response when plants were exposed to drought stress [58]. After being exposed to drought stress, the expression of all other *AdOSCA* genes was upregulated to varying degrees, while *AdOSCA3* was upregulated to 2.9 times the control at 24 h and 2.6 times the control at 48 h. The expression of *AdOSCA9* and *AdOS-CA13* of the kiwifruit *OSCA* family was significantly downregulated at 48 h of drought treatment [42]. While the expression of *MtOSCAs*, a *Tribulus Terrestris OSCA* gene, was not altered significantly following drought treatment [44], the expression of 11 members of the *CsOSCAs* in tea trees showed upregulation under drought stress [43]. The study found that the expression levels of *OSCA* genes in leaves and rhizomes varied in response to dryness and high temperatures and that the expression levels of *NofOSCAs* varied in different tissues, indicating great tissue specificity. Similar to the differential expression of Tribulus Terrestris alfalfa *OSCA* genes in different tissues, the response of *OSCA* genes in leaves and rhizomes to drought and high-temperature stress also varied, suggesting that the response level of *N. franchetii OSCA* genes was different under the stress of different conditions [44].

As a medicinal plant endemic to the Tibetan Plateau and neighboring high-altitude mountainous regions, the structural features and expression patterns of the *OSCA* gene family of *N. franchetii* show multi-level adaptations to the alpine environment. A small number of NofOSCAs are found in chloroplasts or are involved in the regulation of calcium homeostasis of organelles under light stress and low temperature, such as more than three light-responsive *cis*-acting regulatory elements upstream of the promoter of *NofOSCA11*, which is extremely sensitive to changes in light conditions. The majority of NofOSCAs are found in the plasma membrane, which can directly sense changes in cellular osmotic pressure caused by drought, high temperature, low temperature, and other stresses. It is hypothesized that during low-temperature stress, the *NofOSCAs* can finely regulate their expression patterns and participate in Ca^2+^ signaling, transduction, and antifreeze gene regulation. The promoter is rich in methyl jasmonate, abscisic acid, drought, and low-temperature response elements, which can integrate hormonal and abiotic stress signals to dynamically respond to variable stresses in alpine environments. Examples of these include the presence of low-temperature-responsive *cis*-acting regulatory elements upstream of the 22-member promoter. Low-temperature tests can be used in the future to better evaluate the role of *NofOSCAs* in low-temperature adaptation. The expression patterns varied by tissue, with above-ground parts displaying a “first increase followed by a second decrease” or alternating changes, like leaf *NofOSCA7/11*, which balanced transpiration and photosynthesis by regulating stomatal conductance in stages, reflecting the stress response to stress, and roots displaying a continuous increase or a first increase followed by a second decrease, like *NofOSCA6*, which peaked at 24 h of drought and may enhance root osmotic regulation. Moreover, photosynthesis illustrates a delicate balance of work in the stress response.

## 4. Materials and Methods

### 4.1. Experimental Materials and Treatments

Prof. Li Zhonghu of Northwestern University’s College of Life Sciences identified the seedlings as *N. franchetii* after they were collected from Qinghai Province in October 2024. This experiment used the Chinese endemic *N. franchetii* with medicinal value as the test material. In order to maintain the soil’s moisture content and viscosity, the roots were grown in pots at 22.8 °C and 16.8 h of light per day. Watering was carried out every five days. The plants were chosen to experience drought and high-temperature stress after six weeks of re-germination. The water potential (ψw) of the solution could be precisely controlled by varying the concentration of PEG 6000, an inert polymer with a high molecular weight (roughly 6000 Da) that was shown to be unable to penetrate the cell membrane and enter the cell interior. Instead, it only created osmotic potential in the extracellular matrix, simulating the environment of water loss brought on by drought in the soil.

In most cases, the water potentials of a 40% (*w*/*v*) solution of PEG 6000 matched those of a typical drought. Inducing a normal drought response and preventing direct cellular damage from chemical toxicity, a 40% (*w*/*v*) solution of PEG 6000 usually equates to a water potential comparable to the moderate to severe water stress encountered by plant roots under natural drought conditions [59,60]. This characteristic guarantees that a water deficit solely brings on stress effects and prevents direct cell damage from chemical toxicity. Drought stress and PEG are frequently related and can cause comparable cell damage [59].

### 4.2. Identification, Physicochemical Characterization, and Subcellular Localization Prediction of N. franchetii OSCA Family Members

*N. franchetii’s* genome was acquired in the lab (Northwest University, Shaanxi, China), and Novogene Bioinformatics Ltd., Beijing, China). used a paired-end (PE) 150 bp sequencing approach to sequence and analyze the entire genomic DNA using the Illumina NovaSeq 6000 sequencing technology. The TAIR database [61] provided the protein sequences of 15 OSCAs from *A. thaliana*, and the Pfam database (http://pfam.xfam.org/, accessed on 2 September 2024) [62] provided the hmm model RSN1_7TM (PfamID: PF02714) of the OSCA gene family. Candidate genes were submitted to structural domain detection by NCBI-CDD, and those with the RSN1_7TM structural domain were identified as OSCA family members [63]. A bidirectional Blastp comparison was carried out using TBtools (version 2.225) software to retrieve potential members [64].

TMHMM predicted the transmembrane structural domains [65], and they were identified using the WoLF PSORT online website (https://wolfpsort.hgc.jp/, accessed on 13 September 2024) to predict the subcellular localization of *N. franchetii* OSCA proteins [66]. TBtools software was used to analyze the physicochemical data, including the amino acid number, isoelectric point (pI), and molecular weight (MW) of the products encoded by the species.

### 4.3. Conserved Motifs, Conserved Structural Domains, Gene Structure Analysis, and Cis-Acting Element Analysis

The online tool MEME (https://meme-suite.org/meme/tools/meme, accessed on 21 September 2024) was used to mine the conserved motifs of the *N. franchetii* OSCA protein [67], and NCBI-CDD was used to analyze the conserved structural domains [63]. The *cis*-acting element 3000 bp upstream of the *N. franchetii* promoter [68] was analyzed using the plantCARE (http://bioinformatics.psb.ugent.be/webtools/plantcare/html, accessed on 22 September 2024) online website, and TBtools was used to analyze the intron–exon structure of the *N. franchetii OSCA* gene family [64].

### 4.4. Predictive Analysis of Protein Structure

The SOPMA (NPS@:SOPMA secondary structure prediction (https://npsa.lyon.inserm.fr/, accessed on 25 September 2024)) online tool was used to analyze the secondary structures of OSCA proteins in *N. franchetii* [69], and the SWISS-MODEL (SWISS-MODEL Interactive Workspace (https://swissmodel.expasy.org/, accessed on 6 October 2024)) online website was used to predict their tertiary structures [70].

### 4.5. Phylogenetic Analysis of the OSCA Family of N. franchetii

In order to identify the OSCA protein sequences, the laboratory provided the genomic and protein data of *N. franchetii*. The evolutionary tree of OSCA proteins of *A. thaliana*, *N. franchetii*, and *N. incisum* was constructed using the MEGA 11 software [71]. The protein sequences were compared using the ClustalW method of multiple sequences [72], and the maximum-likelihood method was used to construct the evolutionary tree. The resulting image of the evolutionary tree was visualized using the MEGA 11 tool [71] and enhanced on the Evolview v3 website (accessed on 13 October 2024) [73,74,75].

### 4.6. Chromosomal Localization and Covariance Analysis of N. franchetii OSCA Family Members

The *N. franchetii* genome annotation files were acquired in the lab, and TBtools software was used to extract the gene density files through GFF annotation files [64], create chromosomal localization maps, and name the *OSCA* genes in order of their chromosome positions. The One Step MCScanX plug-in in TBtools software was used to conduct an intraspecific covariance study of the *OSCA* gene family of *N. franchetii* [64,76].

### 4.7. Ka/Ks Calculator

The Simple Ka/Ks Calculator (NG) program in the TBtools software was used to determine the synonymous mutation frequency (Ks), non-synonymous mutation frequency (Ka), and Ka/Ks of the covariate gene pairs in order to examine the selection pressure during the gene evolution of NofOSCAs.

### 4.8. GO, KEGG Enrichment, and PPI Networks Analysis

The EggNOG-mapper was used to obtain the functional annotation data for the OSCA gene (http://eggnog-mapper.embl.de/, accessed on 2 April 2025). Lastly, TBtools was used to acquire the enrichment analysis results [64]. Functional interaction network models of OSCA proteins were predicted using the STRING (https://string-db.org/, accessed on 11 October 2024) tool; a score cutoff of 0.40 was required

### 4.9. Analysis of Stress Response Patterns Based on qRT-PCR

The SteadyPure Plant RNA Extraction Kit (Hunan Acres Bioengineering Co., Ltd., AG21019, Hunan, China) was used to extract the total plant RNA. Agarose gel electrophoresis (1.0%) and an ultramicro spectrophotometer (NanoDpro 2000(Kaiao Technology Development Co., Ltd., Beijing, China)) were used to determine the integrity and concentration of the isolated RNA. The Hifair^®^ Ⅲ 1st Strand cDNA Synthesis SuperMix for qPCR (gDNA digester plus) Reverse Transcription Kit (Next Sense Biotechnology Co., Ltd., Shanghai, China 11141ES60) was used to convert the RNA into cDNA. We generated the primer sequences for the *OSCA* gene and the internal reference gene Actin8, and DynaPro Bioscience (Xi’an, China) manufactured them. Three biological replicates of each sample were established for the qRT-PCR analysis of the incomplete *OSCA* genes using the Hieff^®^ qPCR SYBR Green Master Mix (No Rox) Fluorescence Quantification Kit (Next Sense Biotech Co., Ltd., Shanghai, China, 11201ES08). The 2-ΔΔCT method [77] was used to calculate the relative expression of each gene, and OriginPro 2024 (Seifert, 2014) software [78] was used to perform significance analysis using Tukey’s test in a multi-column format, where each column represented a group, and each data column was a sample from that group. For two-by-two comparisons of all groups, the differences in means, standard errors (SEs), 95% confidence intervals, and adjusted *p*-values were listed after a one-way ANOVA was conducted on the tabulated data, and F-values and *p*-values were examined to make sure *p* < 0.05. The findings were plotted using GraphPad Prism 10 for plotting, and the adjusted *p* < 0.05 of the two groups indicated that the means of the two groups were substantially different [79].

### 4.10. Expression Pattern Analysis

To learn more about the expression patterns of *NofOSCA* members, the transcriptome data of *N. franchetii* were examined. RNA sequencing was used to gather transcriptome data of *NofOSCA* members in three tissues (root, leaf, and flower) to analyze their expression profiles. The raw data were filtered using fastp (version 0.23.4), utilizing the genome of *N. incisum* as a reference. SAMtools (version 1.17) was used to exclude unsuccessful comparisons, and Hisat2 (version 2.2.0) was used for comparisons [80,81]. For single-sample assembly and quantification, stringTie (version 2.2.3) was utilized [82]. The TPM values (transcripts per million reads) of OSCA genes were calculated using the RPKM/FPKM or TPM Calculator plugin in TBtools and logarithmically plotted in a heat map.

## 5. Conclusions

In this study, we systematically analyzed the structural characteristics, systematic evolution, and abiotic stress response patterns of the *N. franchetii OSCA* gene family, identified 29 family members from its genome, and found that its promoter region was enriched in a wide range of adversity response elements, such as methyl jasmonate, abscisic acid, drought, and low temperature. The importance of this family in adjusting to high temperatures through the processes of tissue-specific regulation, stress perception, integration of multiple signaling pathways, and plasma membrane localization was further demonstrated by the different members’ unique expression patterns in the roots, stems, and leaves. The importance of this family in alpine environment adaptation is demonstrated through the mechanisms of tissue-specific regulation, stress sensing, integration of several signaling pathways, and plasma membrane localization. Some of the genes that were specifically expressed were found to be either regulated by tissue-specific regulation or potentially play important roles in the functional differentiation of various *N. franchetii* tissues. This analysis of *NofOSCA* expression in various tissues yielded significant candidate genes for resolving the plant’s growth, development, and metabolic mechanisms.

The foundation for molecular breeding of endangered medicinal plants for stress resistance and sustainable resource use is laid by this study, which also closes the gap in *OSCA* gene functional analysis in alpine medicinal plants and offers new insights into the molecular mechanism of *N. franchetii*’s adaptation to the harsh conditions of the Tibetan Plateau, such as drought and high temperature. At the same time, it serves as a reference for the excavation of stress-resistant genes in other crops. Future studies could concentrate on integrating multi-stress response mechanisms, deep mining evolutionary adaptation, translating anti-stress technology, functionally verifying and analyzing key genes’ signaling pathways under light and low temperature stress, etc. We can advance the molecular foundation of alpine plant stress tolerance by using transgenic technology, gene editing, and population genetics studies to further uncover the regulatory network of *OSCA* genes under complex stress and their cooperative development with the Tibetan Plateau environment. Through population genetic analysis, gene editing, and transgenic technology, the molecular basis of *OSCA* genes and their cooperative evolution with the Qinghai–Tibet Plateau environment will be further uncovered, advancing the study and breeding of alpine plants’ stress tolerance mechanisms.

## Figures and Tables

**Table 1:** Physicochemical properties and subcellular localization results of *N. franchetii* OSCA proteins.

Gene ID	Sequence ID	Number of Amino Acids	Molecular Weight	Theoretical pI	Instability Index	Aliphatic Index	Grand Average of Hydropathi-City	Transmembrane Domain	Subcellular Localization
*NofOSCA* *01*	evm.model.Chr01.3154	141	15,918.53	9.37	38.64	104.47	−0.077	0	chloroplast
*NofOSCA* *02*	evm.model.Chr01.5771	761	86,236.02	9.02	44	100.96	0.22	10	plasma membrane
*NofOSCA* *03*	evm.model.Chr01.8116	769	87,453.56	9.23	39.54	103.2	0.159	8	plasma membrane
*NofOSCA* *04*	evm.model.Chr01.8913	709	80,504.33	9.14	42.24	107.38	0.329	9	plasma membrane
*NofOSCA* *05*	evm.model.Chr01.8937	1039	117,120.39	5.87	45.85	95.45	0.04	8	nucleus
*NofOSCA* *06*	evm.model.Chr02.1166	780	88,102.16	9.08	47.05	98.71	0.246	10	plasma membrane
*NofOSCA* *07*	evm.model.Chr03.1679	719	81,000.99	9.28	29.33	103.69	0.286	9	plasma membrane
*NofOSCA* *08*	evm.model.Chr03.1823	887	101,425.11	9.22	45.8	105.64	0.173	12	plasma membrane
*NofOSCA* *09*	evm.model.Chr03.1857	749	85,843.52	9.18	45.24	102.59	0.133	9	cell wall
*NofOSCA* *10*	evm.model.Chr03.4191	719	81,000.99	9.28	29.33	103.69	0.286	9	plasma membrane
*NofOSCA* *11*	evm.model.Chr04.1948	1244	140,334.56	5.67	50.56	89.34	−0.106	2	chloroplast
*NofOSCA* *12*	evm.model.Chr04.1949	756	86,055.52	8.75	40.45	108.7	0.213	10	plasma membrane
*NofOSCA* *13*	08_QHevm.model.Chr05.38_R0	813	91,784.42	6.66	41.19	102.62	0.147	9	plasma membrane
*NofOSCA* *14*	evm.model.Chr05.4066	772	88,535.84	8.9	45.36	101.31	0.151	8	plasma membrane
*NofOSCA* *15*	evm.model.Chr06.115	683	78295.34	9.37	41.1	107.06	0.302	8	plasma membrane
*NofOSCA* *16*	evm.model.Chr06.1871	603	69,265.51	8.99	41.25	97.5	0.023	6	plasma membrane
*NofOSCA* *17*	evm.model.Chr06.5147	760	86,060.78	8.89	37.78	104.33	0.242	10	plasma membrane
*NofOSCA* *18*	evm.model.Chr06.860	751	84,489.39	8.45	43.01	99.68	0.254	11	nucleus
*NofOSCA* *19*	evm.model.Chr07.27	589	67,484.38	9.27	30.79	104.6	0.201	7	plasma membrane
*NofOSCA* *20*	evm.model.Chr07.3709	723	82,372.78	9.05	46.42	108.27	0.188	11	plasma membrane
*NofOSCA* *21*	evm.model.Chr09.276	652	73,488.58	9	33.51	101.49	0.175	8	plasma membrane
*NofOSCA* *22*	evm.model.Chr09.307	652	73,488.58	9	33.51	101.49	0.175	8	plasma membrane
*NofOSCA* *23*	evm.model.Chr10.604	868	99,515.12	8.98	45.95	93.81	−0.043	8	nucleus
*NofOSCA* *24*	evm.model.Contig1135.1	335	37,935.67	8.46	54.76	107.7	0.267	3	plasma membrane
*NofOSCA* *25*	evm.model.Contig219.2	760	86,088.83	8.89	37.78	104.58	0.245	10	plasma membrane
*NofOSCA* *26*	evm.model.Contig25.2	769	87,467.58	9.23	39.29	103.2	0.159	8	plasma membrane
*NofOSCA* *27*	evm.model.Contig2643.1	697	79,801.41	8.04	45.04	100.59	0.119	8	plasma membrane
*NofOSCA* *28*	evm.model.Contig7.67	769	87,313.84	8.35	43.53	100.36	0.263	10	plasma membrane
*NofOSCA* *29*	evm.model.Contig84.18	760	86,088.83	8.89	37.78	104.58	0.245	10	plasma membrane

**Table 2:** Results of two-dimensional structure analysis of *N. franchetii* OSCA proteins.

Protein	Alpha Helix	Extended Strand	Beta Turn	Random Coil
NofOSCA01	39.01	10.64	2.13	48.23
NofOSCA02	49.41	10.51	1.45	38.63
NofOSCA03	51.76	10.01	1.43	36.8
NofOSCA04	57.4	11.85	1.27	29.48
NofOSCA05	44.08	8.28	0.77	46.87
NofOSCA06	49.23	9.62	1.15	40
NofOSCA07	55.77	11.27	1.25	31.71
NofOSCA08	50.39	10.37	1.69	37.54
NofOSCA09	55.01	10.81	1.07	33.11
NofOSCA10	55.77	11.27	1.25	31.71
NofOSCA11	25.64	18.81	4.66	50.88
NofOSCA12	52.38	10.98	1.32	35.32
NofOSCA13	48.95	10.58	1.35	39.11
NofOSCA14	50.91	10.49	1.3	37.31
NofOSCA15	54.76	10.98	1.9	32.36
NofOSCA16	49.92	10.78	1	38.31
NofOSCA17	52.11	10.26	1.71	35.92
NofOSCA18	51.26	10.12	1.2	37.42
NofOSCA19	59.08	10.87	1.02	29.03
NofOSCA20	54.5	10.93	1.24	33.33
NofOSCA21	55.67	11.2	1.23	31.9
NofOSCA22	55.67	11.2	1.23	31.9
NofOSCA23	45.74	9.1	1.04	44.12
NofOSCA24	53.43	8.36	1.79	36.42
NofOSCA25	52.11	10.26	1.71	35.92
NofOSCA26	51.5	10.79	1.17	36.54
NofOSCA27	51.79	11.91	1.58	34.72
NofOSCA28	50.72	9.75	1.17	38.36
NofOSCA29	52.11	10.26	1.71	35.92

## Data Availability

All data are contained within the article and the Supplementary Materials.

## Supplementary Materials

The following supporting information can be downloaded at: https://www.mdpi.com/article/10.3390/ijms26115043/s1.
